# Association of fecal short-chain fatty acids with clinical severity and gut microbiota in essential tremor and its difference from Parkinson’s disease

**DOI:** 10.1038/s41531-023-00554-5

**Published:** 2023-07-17

**Authors:** Pei Huang, Pingchen Zhang, Juanjuan Du, Chao Gao, Jin Liu, Yuyan Tan, Shengdi Chen

**Affiliations:** 1 Department of Neurology and Institute of Neurology, Ruijin Hospital, Shanghai Jiao Tong University School of Medicine, 200025 Shanghai, China; 2 Lab for Translational Research of Neurodegenerative Diseases, Shanghai Institute for Advanced Immunochemical Studies (SIAIS), Shanghai Tech University, 201210 Shanghai, China

**Keywords:** Parkinson's disease, Diagnostic markers, Constipation, Neurodegeneration

## Abstract

Diagnosis of essential tremor (ET) at an early stage can be difficult, especially when distinguishing it from healthy controls (HCs) and Parkinson’s disease (PD). Recently, stool sample analysis of gut microbiota and its metabolites provides new ways to detect novel biomarkers for neurodegenerative diseases. Short-chain fatty acids (SCFAs), as the main metabolites of gut microbiota, were reduced in the feces of PD. However, fecal SCFAs in ET have never been investigated. We aimed to investigate the fecal SCFA levels in ET, assess their relationships with clinical symptoms and gut microbiota, and identify their potential diagnostic abilities. Fecal SCFAs and gut microbiota in 37 ET, 37 de novo PD and 35 HC were measured. Constipation, autonomic dysfunction and tremor severity were evaluated by scales. ET had lower fecal propionic, butyric and isobutyric acid levels than HC. Combined propionic, butyric and isobutyric acid distinguished ET from HC with an AUC of 0.751 (95% CI: 0.634–0.867). ET had lower fecal isovaleric and isobutyric acid levels than PD. Isovaleric and isobutyric acid differentiated ET from PD with an AUC of 0.743 (95% CI: 0.629–0.857). Fecal propionic acid was negatively correlated with constipation and autonomic dysfunction. Isobutyric and isovaleric acid were negatively associated with tremor severity. Lowered fecal SCFAs were related to a decreased abundance of *Faecalibacterium* and *Catenibacterium* in ET. In conclusion, fecal SCFAs were decreased in ET and correlated with clinical severity and gut microbiota changes. Fecal propionic, butyric, isobutyric and isovaleric acid might be potential diagnostic and differential diagnostic biomarkers for ET.

## Introduction

Essential tremor (ET) is a progressive, chronic, neurodegenerative disease mainly characterized by an action tremor of the upper limbs, possibly with the involvement of other parts of the body, such as the head, vocal cords and lower limbs^1^. The clinical profile of ET is characterized by not only motor symptoms but also some nonmotor features, including gastrointestinal disorders^2^. Numerous studies have been performed to investigate the pathological and physiological features of essential tremor; however, a clear pathophysiological mechanism has not been identified^3,4^. Recent studies have shown that dysfunction of the microbiota-gut–brain axis might lead to neurodegenerative diseases, and increasing evidence has indicated a potential bidirectional relationship between gut microbiota and neurodegenerative diseases^5,6^. It was notable that fecal microbiota transplantation simultaneously ameliorated the patient’s essential tremor and irritable bowel syndrome in a case report, which might suggest a close link between gut microbiota and essential tremor^7^. Furthermore, we identified specific gut microbiota alterations in ET patients, which strongly demonstrated an important role of gut dysbiosis in ET^8^.

Regarding gut dysbiosis in neurodegenerative diseases, PD is the most widely studied^5^. The imbalanced microbiome enhances intestinal permeability and activates enteric glial cells, which contribute to α-synucleinopathy^9–11^. PD shares certain overlapping features with ET, such as a similar tremor frequency, overlapping resting tremors (a typical PD tremor), and postural tremors (mainly in ET patients) in both ET and PD patients, making it challenging to distinguish one from the other at an early stage^12^. Thus, it is urgent for us to open a helpful window for distinguishing ET from PD. In this context, studying the specific gut dysbiosis and related metabolite changes in ET and identifying its differences from PD might provide potential biomarkers for ET diagnosis and differential diagnosis.

Short-chain fatty acids (SCFAs) are the main metabolites produced by gut bacterial fermentation of dietary fiber and are speculated to be pivotal in gut–brain cross talk^13,14^. SCFAs are absorbed in colonocytes and transported to the liver via the portal venous system, and some SCFAs enter the systemic circulation. SCFAs have local effects in maintaining intestinal barrier integrity and shaping gut mucosal innate immunity^15^. They also have remote effects on the blood–brain barrier (BBB) by promoting tight junction proteins and crossing the BBB to activate neurons through stimulation of G protein-coupled receptors (GPCRs)^16^. Acetic acid, propionic acid and butyric acid are the most abundant SCFAs in the colon. Previous studies have identified reduced fecal levels of acetic acid, propionic acid and butyric acid in PD patients^17^. However, the fecal SCFA levels in ET patients have never been investigated.

Thus, our study aimed to identify specific fecal SCFA alterations in ET patients and their differences from PD patients, to assess the relationships of fecal SCFAs with clinical symptoms and gut microbiota in ET and to identify the potential diagnostic and differential diagnostic ability of fecal SCFAs. To eliminate the confounding factor of anti-PD medication, we selected de novo PD patients as disease controls.

## Results

### Demographics and clinical characteristics of subjects

The demographics and clinical characteristics of 37 ET, 37 PD and 35 HC are summarized in Table 1. ET, PD and HC were matched in age, sex and BMI. The three groups also had similar percentages of smoking, alcohol, coffee and tea consumption. The PD group showed higher Wexner scores (*P* = 0.004) and HAMD-17 scores (*P* = 0.001) than the HC group, and the ET group had higher HAMA scores (*P* = 0.011) and HAMD-17 scores (*P* = 0.011) than the HC group. The ET group had a significantly longer disease duration than the PD group (*P* < 0.001).

**Table 1:** Demographics and characteristics of the participants.

	HC (*n* = 35)	ET (*n* = 37)	PD (*n* = 37)	*P* value	*P*^1^	*P*^2^	*P*^3^
Male (*n*, %)	19 (54.3%)	16 (43.2%)	18 (48.6%)	0.645	/	/	/
Age (y)	63.086 ± 7.834	62.108 ± 6.879	61.257 ± 6.385	0.618	/	/	/
BMI (Kg/m^2^)	24.145 ± 3.107	23.717 ± 2.672	23.604 ± 1.777	0.644	/	/	/
Smoke (*n*, %)	25 (71.4%)	30 (81.1%)	28 (75.7%)	0.628	/	/	/
Alcohol (*n*, %)	27 (77.1%)	30 (81.1%)	27 (73.0%)	0.709	/	/	/
Tea (*n*, %)	24 (68.6%)	29 (78.4%)	24 (64.9%)	0.420	/	/	/
Coffee (*n*, %)	33 (94.3%)	31 (83.8%)	33 (89.2%)	0.363	/	/	/
SCOPA-AUT	/	3.405 ± 3.004	3.919 ± 4.815	0.584	/	/	/
Disease duration (y)	/	8.324 ± 9.226	1.842 ± 1.670	<0.001	/	/	/
Bristol score	4.257 ± 0.919	3.784 ± 1.031	4.432 ± 1.444	0.049	0.257	1.000	0.053
Wexner score	0.629 ± 1.114	1.541 ± 2.219	2.432 ± 3.167	0.006	0.304	0.004	0.312
MMSE score	28.771 ± 1.516	28.324 ± 1.701	28.460 ± 2.116	0.562	/	/	/
HAMD-17 score	1.171 ± 1.807	3.432 ± 3.633	3.946 ± 3.778	0.001	0.011	0.001	1.000
HAMA score	1.943 ± 2.043	4.027 ± 3.193	3.108 ± 3.454	0.014	0.011	0.299	0.561
MDS-UPDRS score	/	/	29.541 ± 19.245	/	/	/	/
MDS-UPDRS III score	/	/	20.081 ± 13.011	/	/	/	/
H-Y score	/	/	1.446 ± 0.483	/	/	/	/
FTM score	/	9.054 ± 10.499	/	/	/	/	/
TETRAS score	/	13.108 ± 9.815	/	/	/	/	/

*ET* Essential Tremor, *PD* Parkinson’s disease, *HC* healthy control, *BMI* body mass index, *MDS-UPDRS* Movement Disorder Society sponsored version of the Unified Parkinson’s Disease Rating Scale, *MMSE* Mini Mental State Examination, *HAMD-17* Hamilton Depression Scale-17 items, *HAMA* Hamilton Anxiety Scale, *H-Y* Hoehn and Yahr stage, *SCOPA-AUT* Scale for Outcomes in Parkinson’s Disease for Autonomic Symptoms, *FTM* Fahn-Tolosa-Marin Clinical Rating Scale for Tremor, *TETRAS* Tremor Research Group (TRG) Essential Tremor Rating Assessment Scales.

*P*^1^: HC vs. ET, *P*^2^: HC vs. PD, *P*^3^: ET vs. PD.

### Between-group differences in fecal levels of SCFAs

**Fig. 1:**
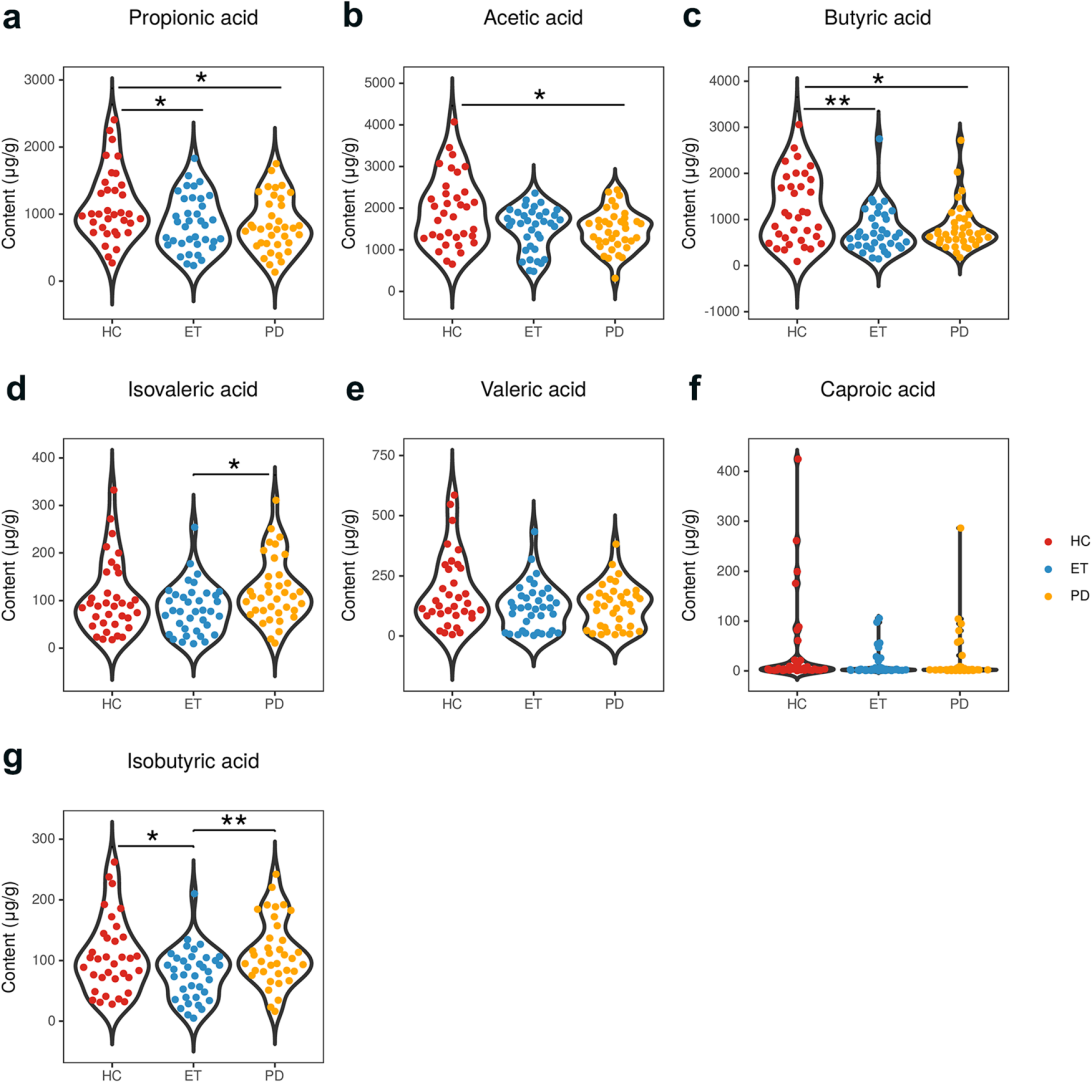
Between-group differences in fecal levels of SCFAs. **a**–**g** represent group comparisons of propionic acid, acetic acid, butyric acid, isovaleric acid, valeric acid, caproic acid, and isobutyric acid, respectively. The three groups were significantly different in the fecal levels of propionic acid, acetic acid, butyric acid, isovaleric acid and isobutyric acid. ET essential tremor, PD Parkinson’s disease, HC healthy control, SCFAs short-chain fatty acids. Significant differences are indicated by *P < 0.05 and **P < 0.01.

The three groups were significantly different in the fecal levels of propionic acid (*P* = 0.023), acetic acid (*P* = 0.039), butyric acid (*P* = 0.020), isovaleric acid (*P* = 0.045) and isobutyric acid (*P* = 0.015). In further post hoc analysis, the ET group showed significantly lower levels of propionic acid (*P* = 0.023), butyric acid (*P* = 0.007) and isobutyric acid (*P* = 0.040) than the HC group. ET patients had lower levels of isovaleric acid (*P* = 0.014) and isobutyric acid (*P* = 0.005) than PD patients. Moreover, PD patients had lower fecal levels of propionic acid (*P* = 0.013), acetic acid (*P* = 0.016) and butyric acid (*P* = 0.041) than HCs (Fig. 1 and Supplementary Table 1).

Considering the difference in disease duration between the ET and PD groups, we screened 33 early PD patients and 16 ET patients (disease duration ≤3 y) for further comparison (Supplementary Table 2). The results showed that ET had significantly lower propionic acid in feces than HC (*P*= 0.015). Differences between ET and HC in butyric acid and isobutyric acid were not significant but still had a trend (*P* = 0.082). ET patients had significantly decreased fecal levels of isobutyric acid compared with PD patients (*P* = 0.030). The difference between ET and PD in isovaleric acid was not significant but still had a trend (*P* = 0.084). PD patients were significantly lower in propionic acid (*P* = 0.023), acetic acid (*P* = 0.020), and butyric acid (*P* = 0.044) than HCs. These results (Supplementary Fig. 1) were generally consistent with the primary results. Differences between the results of the total sample set and early patient subset might be related to the small samples in the subset, leading to a decrease in data statistical efficiency.

**Fig. 2:**
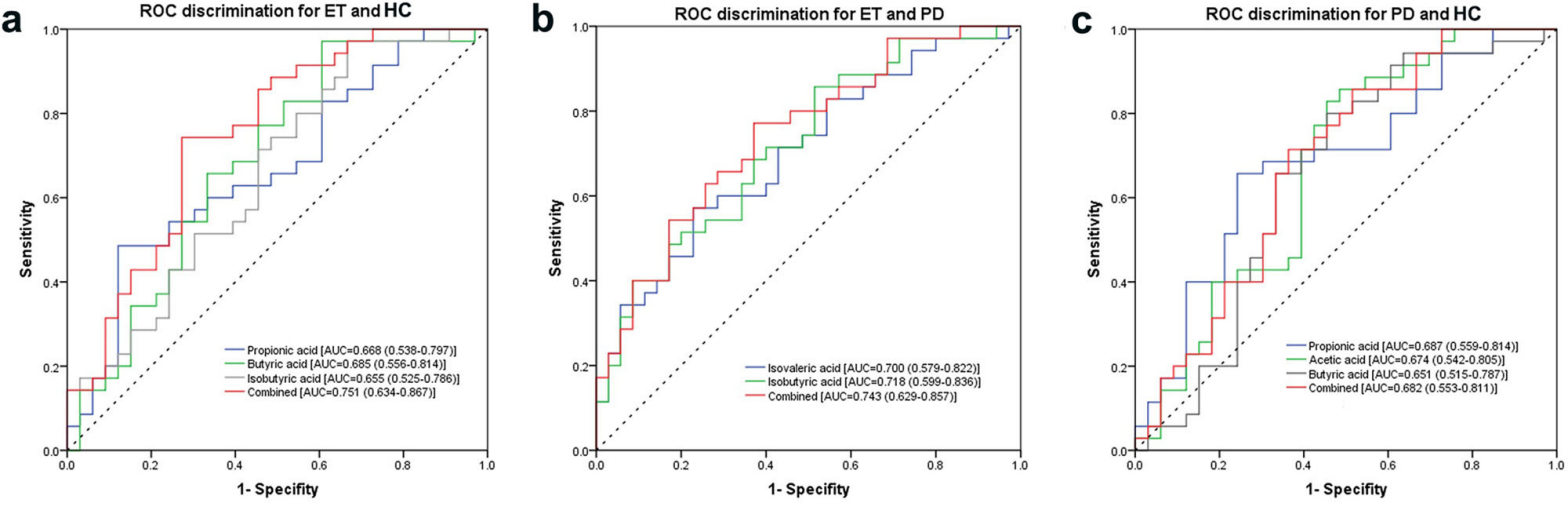
Differential fecal concentrations of SCFAs performed as ROC curves of discriminators between groups. **a** ROC discrimination for ET and HC; **b** ROC discrimination for ET and PD; **c** ROC discrimination for PD and HC. ET essential tremor, PD Parkinson’s disease, HC healthy control, SCFAs short-chain fatty acids.

We further examined whether fecal levels of SCFAs distinguished ET patients from HCs or PD patients. Based on the ROC analysis, the difference in propionic acid level had an AUC of 0.668 (95% CI: 0.538–0.797) to distinguish ET patients from HCs. ET patients could be separated from HCs by butyric acid level with an AUC of 0.685 (95% CI: 0.556–0.814). Isobutyric acid level differences could discriminate ET patients from HCs with an AUC of 0.655 (95% CI: 0.525–0.786). By combining propionic acid, butyric acid and isobutyric acid levels, a higher AUC of 0.751 was obtained (95% CI: 0.634–0.867) with 74.3% sensitivity and 72.9% specificity (Fig. 2a). For the distinction between ET and PD patients, the isovaleric acid level had an AUC of 0.700 (95% CI: 0.579–0.822), and the isobutyric acid level had an AUC of 0.718 (95% CI: 0.599–0.836). The combination of isovaleric acid and isobutyric acid levels had a higher AUC of 0.743 (95% CI: 0.629–0.857) with 74.3% sensitivity and 62.9% specificity (Fig. 2b). Furthermore, we examined whether fecal levels of SCFAs distinguished PD patients from controls. Based on the ROC analysis, the identification of PD patients by differences in propionic acid levels had an AUC of 0.687 (95% CI: 0.559–0.814) with 68.6% sensitivity and 68.7% specificity. Acetic acid level differences could discriminate PD patients from HCs with an AUC of 0.674 (95% CI: 0.542–0.805). With only butyric acid levels, the PD patients could be separated from HCs with an AUC of 0.651 (95% CI: 0.515–0.787). By combining propionic acid, acetic acid and butyric acid levels, an AUC of 0.682 was obtained (95% CI: 0.553–0.811) (Fig. 2c).

### Correlations between fecal SCFA levels and clinical measurements

**Fig. 3:**
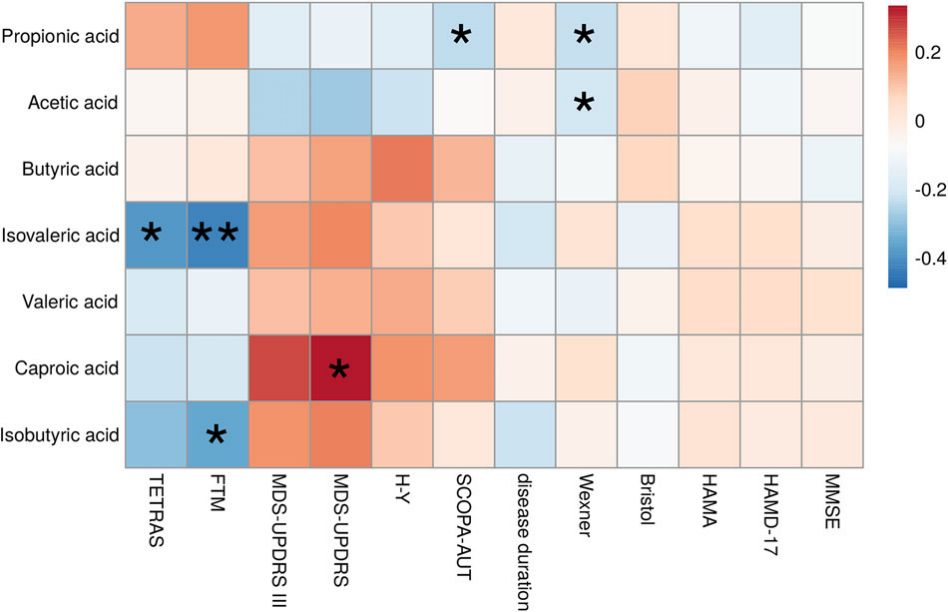
Heat maps representing the Spearman correlation of fecal SCFAs and clinical features. Fecal levels of isobutyric acid were negatively correlated with FTM scores, isovaleric acid was negatively associated with FTM and TETRAS scores, propionic acid was negatively correlated with SCOPA-AUT scores, caproic acid was positively associated with MDS-UPDRS scores, and propionic acid and acetic acid were both negatively correlated with Wexner scores. MDS-UPDRS Movement Disorder Society sponsored version of the Unified Parkinson’s Disease Rating Scale, MMSE Mini Mental State Examination, HAMD-17 Hamilton Depression Scale-17 items, HAMA Hamilton Anxiety Scale, H-Y Hoehn and Yahr stage, SCFAs Short-Chain Fatty Acids, SCOPA-AUT Scale for Outcomes in Parkinson’s Disease for Autonomic Symptoms, FTM Fahn-Tolosa-Marin Clinical Rating Scale for Tremor, TETRAS Tremor Research Group (TRG) Essential Tremor Rating Assessment Scale. Significant differences are indicated by *P < 0.05 and **P < 0.01.

Among ET patients, fecal levels of isobutyric acid were negatively correlated with FTM scores (*r* = −0.349, *P* = 0.034), and fecal levels of isovaleric acid were negatively associated with FTM scores (*r* = −0.421, *P* = 0.001) and TETRAS scores (*r* = −0.382, *P* = 0.020). Among ET and PD patients, fecal levels of propionic acid were negatively correlated with SCOPA-AUT scores (*r* = −0.236, *P* = 0.043) (Fig. 3 and Supplementary Table 3). Disease duration was not significantly correlated with SCFAs in either the ET group (*P* ≥ 0.161) or the PD group (*P* ≥ 0.246) (Supplementary Table 4). Among PD patients, fecal levels of caproic acid were positively associated with MDS-UPDRS scores (*r* = 0.335, *P* = 0.042). Among all the participants, fecal levels of propionic acid (*r* = −0.230, *P* = 0.016) and acetic acid (*r* = -0.210, *P* = 0.029) were both negatively correlated with Wexner scores (Fig. 3 and Supplementary Table 3).

### Gut microbiome species correlate with fecal levels of SCFAs

We further explored the discriminant gut microbiota using LEfSE analysis, and the genus level of the relative abundance data was selected for further analysis. Comparisons were performed between ET and HC and between ET and PD. Next, Spearman correlation analyses were performed between the relative abundance of gut microbiota and fecal levels of SCFAs among the two comparison groups.

**Fig. 4:**
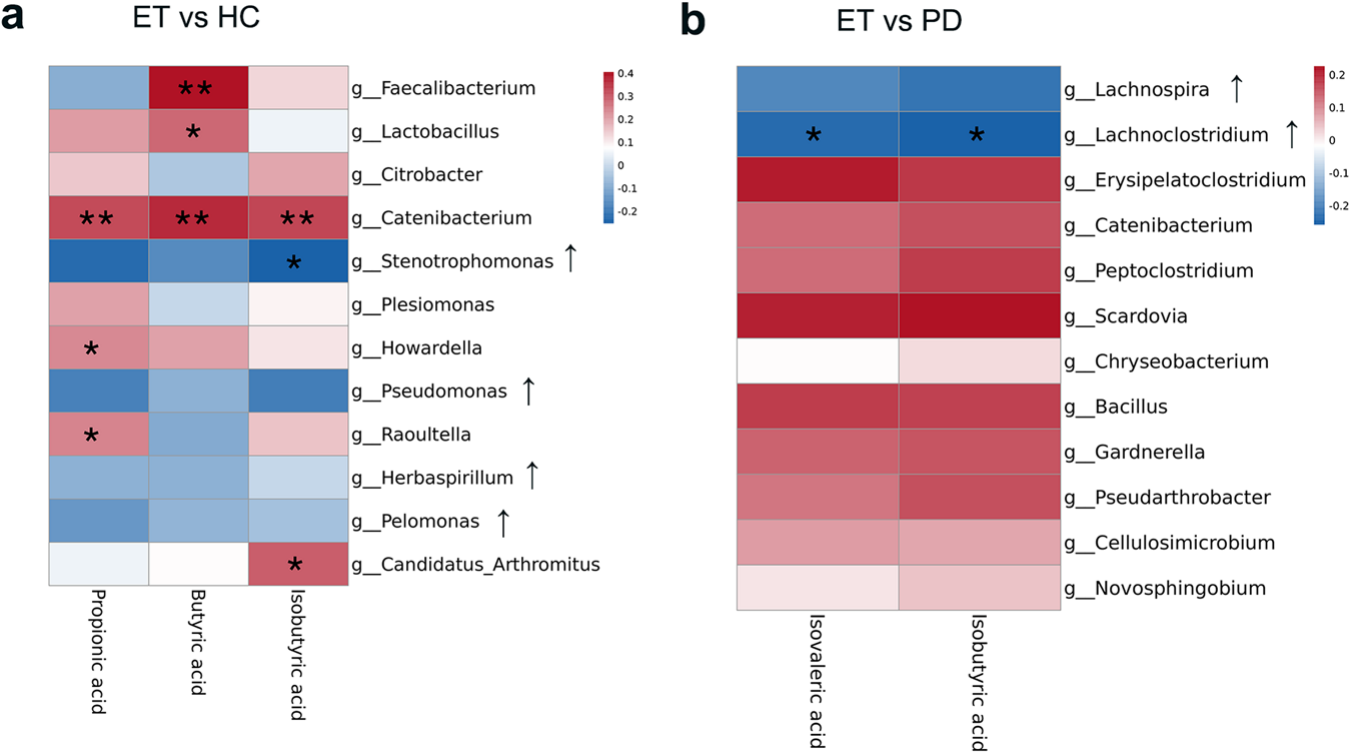
Heat maps representing the correlation of fecal microbiota at the genus level and SCFAs. **a** Correlation analysis in ET and HC. The abundances of *Faecalibacterium* (positively correlated with butyric acid) and *Catenibacterium* (positively correlated with propionic, butyric and isobutyric acid) were found to be decreased in ET and positively correlated with the fecal levels of SCFAs after FDR adjustment. **b** Correlation analysis of ET and PD. No significant association was found after FDR adjustment. ET essential tremor, PD Parkinson’s disease, HC healthy control, SCFAs short-chain fatty acids. Significant differences are indicated by *P < 0.05 and **P < 0.01.

In the analysis of ET vs HC, the abundances of *Faecalibacterium* (correlated with butyric acid, *r* = 0.408, *P* < 0.001), *Lactobacillus* (correlated with butyric acid, r = 0.283, *p* = 0.016), *Catenibacterium* (correlated with propionic acid, *r* = 0.327, *P* = 0.005; correlated with butyric acid, *r* = 0.374, *P* = 0.001; correlated with isobutyric acid, *r* = 0.329, *P* = 0.005), *Howardella* (correlated with propionic acid, *r* = 0.242, *P* = 0.041), *Raoultella* (correlated with propionic acid, *r* = 0.249, *P* = 0.035) and *Candidatus Arthromitus* (correlated with isobutyric acid, *r* = 0.302, *P* = 0.010) were found to be decreased in ET and positively correlated with the fecal levels of SCFAs. However, the abundance of *Stenotrophomonas* was increased in ET and negatively correlated with the fecal levels of isobutyric acid (*r* = −0.250, *P* = 0.034). After FDR adjustment, only the correlations between *Faecalibacterium, Catenibacterium* and SCFAs remained significant (*P* ≤ 0.045) (Fig. 4 and Supplementary Table 5).

In the analysis of ET vs PD, the abundance of *Lachnoclostridium* was found to be increased in ET and negatively correlated with the fecal levels of isovaleric acid (*r* = −0.238, *P* = 0.041) and isobutyric acid (*r* = −0.257, *P* = 0.027). After FDR adjustment, either of them remained significant (*P* ≥ 0.295) (Fig. 4 and Supplementary Table 5).

## Discussion

This study is an integrated study examining the fecal levels of SCFAs and linking them to changes in gut microbiota and symptom severity in ET patients compared with HCs and PD patients. We found that fecal levels of SCFAs were decreased in ET patients and correlated with clinical severity and specific gut microbiota changes. The combined fecal levels of SCFAs could distinguish ET from HC and PD.

Compared with HCs, ET patients had lower fecal levels of propionic acid, butyric acid and isobutyric acid. The combination of propionic acid, butyric acid and isobutyric acid could distinguish ET from HC with an AUC of 0.751 (95% CI: 0.634–0.867) with 74.3% sensitivity and 72.9% specificity, indicating their potential roles as diagnostic biomarkers for ET. Further analysis showed that fecal levels of propionic acid were negatively correlated with the Wexner score and SCOPA-AUT score. Isobutyric acid levels in feces were negatively associated with FTM scores. On the other hand, the lowered levels of butyric acid in ET were related to the decreased abundance of *Faecalibacterium* and *Catenibacterium*, which are SCFA-producing microbiota. In addition, the decreased abundances of *Catenibacterium* in ET were also correlated with the lowered fecal levels of propionic acid and isobutyric acid.

Most SCFAs produced in the colon are absorbed by colonocytes mainly via H^+^-dependent or sodium-dependent monocarboxylate transporters. The absorbed SCFAs are used as a source of energy for colonocytes, and those that are not metabolized in colonocytes are transported into the portal circulation^18^. SCFAs can impact gut motility, strengthen gut barrier functions, and influence host metabolism and immunity^19^. The concentrations of fecal butyric acid, acetic acid and propionic acid were previously found to be decreased in PD patients compared with HCs^17^, which was consistent with our results. Our study identified reduced SCFAs in ET patients, but little is known about the role of SCFAs in the pathology of ET. Butyric acid and propionic acid could bind to GPCRs and influence GPCR-dependent signaling, such as MAPK and NF-κB signal transduction^20^. A leading concept of the gut–brain axis is that gut microbes secrete SCFAs that could influence host signaling, affecting functions in both the gut and brain. Because butyric acid and propionic acid exhibit strong inhibition of histone deacetylase (HDAC) activity^21^ and butyric acid might also act as a ligand of transcription factors, they have a broad impact on host metabolism, differentiation, and proliferation mainly due to their impact on gene regulation^22^. Based on evidence of SCFAs and neurodegenerative diseases, butyric acid has been implicated as a therapeutic candidate because of its ability to correct the disrupted activity of HDAC, which could mediate dopaminergic neuron death in PD^23–25^. Animal studies have also confirmed the capacity of butyric acid to prevent the degeneration of dopaminergic neurons and ameliorate motor impairments in PD models^26,27^. Propionic acid was found to limit inflammatory responses and protect BBB integrity^28,29^. Studies have shown that propionic acid promotes the survival of dopaminergic neurons against rotenone toxicity in the PD model^30^ and that orally administered propionic acid rescues dopaminergic neuronal loss and motor deficits in PD mice^31^. Little is known about the functions of isobutyric acid. However, a recent study reported that *Bacteroides ovatus* colonization in mice increased the abundance of intestinal SCFAs (including acetic acid, propionic acid, isobutyric acid and isovaleric acid) and the concentrations of intestinal GABA, highlighting connections between gut microbiota and intestinal SCFA/neurotransmitter concentrations^32^. For ET, pathological changes in cerebellar abnormalities include changes to Purkinje cell axons and dendrites, displacement and loss of Purkinje cells, changes to basket cell axonal processes, abnormal distribution of climbing fiber connections to Purkinje cells and changes in GABA receptors in the dentate nucleus, resulting in reduced GABAergic output from the cerebellum^3,4,33^. Whether SCFAs are related to the neurodegeneration of Purkinje cells and the reduction in GABAergic output in the cerebellum remains unknown. Our results indicated a close relationship between SCFAs and ET; however, the cross-sectional study design precludes making any inferences of a causal relationship between SCFAs and the disease process in ET. Further longitudinal follow-up studies with serial measurements of fecal SCFAs as well as animal studies for mechanistic investigations are warranted.

SCFAs are thought to stimulate colonic smooth muscle contractility^34^. The lack of SCFAs aggravated symptoms of constipation, and SCFA supplementation could attenuate constipation symptoms of PD^35^. Our results also demonstrated a significant association between the decrease in fecal SCFAs and the aggravation of constipation and autonomic dysfunction in ET patients. A case report found that microbiota transplantation simultaneously ameliorated patients’ essential tremor and irritable bowel syndrome^7^, which also suggested a close link between gut microbiota and ET. Thus, we considered that fecal SCFAs/microbiota could impact host gut motility and autonomic function.

Lowered fecal levels of SCFAs in ET were found to be associated with decreased abundances of *Faecalibacterium* (correlated with butyric acid) and *Catenibacterium* (correlated with propionic acid, butyric acid, and isobutyric acid). After FDR correction, the relationship remained significant. Both *Faecalibacterium* and *Catenibacterium* are SCFA-producing microbiota. *Faecalibacterium* is known to be the butyrate-producing microbiota^36^ and the major fermentation products of *Catenibacterium* are acetate, butyrate and lactate^37^. *Faecalibacterium* was 100% detected in both the ET and HC groups; the median relative abundance in the ET group was 2.06%, while the median relative abundance in the HC group was 3.28% (LDA 3.870). *Catenibacterium* was detected in 21.6% of the (8/37) HC group but was detected in only one sample of the ET group (1/35). The decrease in *Catenibacterium* and the undetectability of *Catenibacterium* in ET might also indicate its correlation to disease pathogenicity. The median relative abundance of *Catenibacterium* in the HC group was 0.07% (LDA 2.129). In addition, *Lactobacillus* was related to changes in fecal butyric acid (*P* = 0.016, *P* = 0.096 after FDR correction), and *Candidatus Arthromitus* was associated with alterations in isobutyric acid (*P* = 0.016, *P* = 0.072 after FDR correction). After FDR correction, only a trend of correlation remained without statistical significance. *Lactobacillus* is also considered an SCFA (acetic acid, propionic acid, isobutyric acid, butyric acid) producer^38^, while *Candidatus Arthromitus* is a specific inducer of differentiation in T helper 17 (Th17) cells, associated with the immune balance of Th1/2 and Treg/Th17^39^. A recent study demonstrated that the elevation of *Candidatus Arthromitus* in the feces might lead to colon inflammation, gut barrier dysfunction, and systemic inflammation^40^. *Lachnoclostridium* was increased in ET compared to PD. The abundance of *Lachnoclostridium* was found to be negatively correlated with isovaleric acid and isobutyric acid. After FDR adjustment, either of them remained significant (*P* ≥ 0.295). *Lachnoclostridium* is known to be an inflammation-associated bacterium that might lead to gut barrier dysfunction^41^. Our previous study reported gut microbiota changes in ET patients^8^. Here, we further reported SCFA alterations in ET and revealed the relationship between gut dysbiosis and SCFA changes. Decreased levels of SCFAs were closely related to gut dysbiosis and tremor severity in ET. Our results indicated that the gut–brain axis might have an important role in the pathogenesis of ET, but further studies of animal models are warranted.

Compared with PD patients, ET patients had lower fecal levels of isovaleric acid and isobutyric acid. The combination of isovaleric acid and isobutyric acid identified ET from PD with an AUC of 0.743 (95% CI: 0.629–0.857) with 74.3% sensitivity and 62.9% specificity, indicating their potential roles as differential diagnostic biomarkers for ET. The levels of isovaleric acid in feces were negatively correlated with FTM scores and TETRAS scores. Isobutyric acid levels in feces were negatively associated with FTM scores. The lowered levels of isobutyric acid were related to the decreased abundance of *Catenibacterium*. Little is known about the functions of isovaleric and isobutyric acid. A previous study indicated that *Bacteroides ovatus* colonization in mice increased the abundance of intestinal SCFAs (including acetic acid, propionic acid, isobutyric acid and isovaleric acid) and the concentrations of intestinal GABA, highlighting links between gut microbiota and intestinal SCFA/neurotransmitter concentrations^32^. Interestingly, isobutyric acid was observed at similar levels between the PD and HC groups but was distinct between the ET and PD (or HC) groups. Isobutyric acid could distinguish ET from PD with an AUC of 0.718 (95% CI: 0.599–0.836) and identify ET from NC with an AUC of 0.655 (95% CI: 0.525–0.786). In addition, isobutyric acid levels were also related to tremor severity, which further strengthens its association with ET. Whether oral supplementation with isobutyric acid could relieve tremor severity in ET patients is worth future research.

In conclusion, fecal SCFAs were decreased in ET patients and correlated with the clinical severity and specific gut microbiota changes of ET. Fecal propionic acid, butyric acid and isobutyric acid might be diagnostic biomarkers for ET, while isobutyric acid and isovaleric acid might be differential diagnostic biomarkers for ET. Isobutyric acid changes in feces might be more ET-specific than other SCFA alterations.

Our study has several limitations. First, dietary patterns and food preferences may impact microbiota expression, larger-scale research samples from different populations are warranted, and a complete and systematic dietary interrogation, such as the Food Frequency Questionnaire, should be introduced in future studies. Second, the cross-sectional study design precludes making any inferences of a causal relationship between SCFAs and the disease process in ET. Further longitudinal follow-up studies with serial measurements of fecal SCFAs are warranted. Third, the diagnostic and differential diagnostic abilities of fecal levels of SCFAs should be verified by independent samples from ET, HC and PD. Larger numbers of another independent fecal sample should be tested in the future. Finally, the PD patients in our cohort had a significantly shorter disease duration than the ET patients. We mainly matched ET, PD and HC in age, sex and BMI. Considering the difference in disease duration between the ET and PD groups, we also screened 33 early PD patients and 16 ET patients (disease duration ≤3 y) for further comparison. Between-group differences in SCFAs were generally consistent with our primary data. In addition, we did not find a correlation between disease duration and changes in SCFAs. However, it is better to recruit early PD and ET patients with short disease durations to complete the verification with a larger sample in the future.

## Methods

### Standard protocol approvals and patient consents

The research protocol was approved by the Ethics Committee of Ruijin Hospital affiliated with Shanghai Jiao Tong University School of Medicine (RHEC2018–243). Written informed consent was obtained from all of the participants.

### Participants and clinical evaluation

One hundred and nine subjects (37 ET, 37 PD, and 35 HC) from the outpatient clinic of the Movement Disorders Center in Ruijin Hospital affiliated with Shanghai Jiao Tong University School of Medicine were enrolled in our study between January 2019 and December 2022. Inclusion criteria were: (1) aged 25–85 years, (2) ET patients were diagnosed according to MDS Task Force criteria^42^ and PD diagnosis was based on MDS criteria^43^, (3) no anti-PD medication intake before the fecal sample collection of all subjects, (4) solely beta-blocker intake or no relevant medication intake before the fecal sample collection for ET group. HCs matched by age, sex, and body mass index (BMI) were selected simultaneously. Exclusion criteria were: (1) vegetarian, (2) malnutrition, (3) chronic gastrointestinal disorder (including inflammatory bowel disease, gastric or duodenal ulcer), (4) severe chronic illness (including malignant tumor, heart failure, renal insufficiency, hematological disorder, hypertension and diabetes, etc.), (5) history of major gastrointestinal surgery, (6) ongoing or regular consumption of yogurt, (7) use of any probiotic or antibiotic within one month, (8) ongoing use of corticosteroid, proton pump inhibitor, statin, metformin, immunosuppressant or anti-neoplastic medication, and (9) severe cognitive deficit that obstructed the execution of clinical assessment.

All subjects provided information on medical history, weight, and height for the calculation of BMI and accepted neurological examination and clinical assessments, such as the Hamilton Anxiety Scale (HAMA)^44^ for anxiety, Hamilton Depression Scale-17 items (HAMD-17)^45^ for depression, Wexner constipation score^46^ and Bristol stool scale^47^ for constipation severity, and Mini Mental State Examination (MMSE)^48^ for cognition. The Scale for Outcomes in Parkinson’s Disease for Autonomic Symptoms (SCOPA-AUT)^49^ was interrogated for ET and PD patients for autonomic dysfunction. The Fahn-Tolosa-Marin Clinical Rating Scale for Tremor (FTM) and the Tremor Research Group (TRG) Essential Tremor Rating Assessment Scale (TETRAS)^50^ were examined among ET patients; the Movement Disorder Society sponsored version of the Unified Parkinson’s Disease Rating Scale (MDS-UPDRS)^51^ and the Hoehn and Yahr (H-Y)^52^ stage were examined for PD patients.

### Fecal sample collection and SCFA level measurement

Each participant was asked to collect a fecal sample in the morning using fecal collection containers. The containers were transferred to ice and stored at −80 °C prior to processing. The analysis of SCFAs was performed following routine operations by Tinygene Bio-Tech (Shanghai) Co., Ltd. For each subject, 400 mg of fresh fecal samples was applied to the analysis of SCFAs after grinding and sonication pretreatment. Fecal analyses for individual SCFAs were performed with gas chromatography-mass spectrometry (GC-MS) and liquid chromatography tandem MS (LC-MS/MS).

### Gut microbiota analyses and sequencing

The DNA was extracted from 200 mg samples using the QIAamp® Fast DNA Stool Mini Kit (QIAGEN, Hilden, Germany) following the manufacturer’s instructions. Microbial composition was determined by 16 S rRNA gene sequencing of DNA extracted from stool by amplifying the V3–V4 regions. DNA was checked by running the samples on 1.2% agarose gels. Polymerase chain reaction (PCR) amplification of 16 S rRNA genes was performed using general bacterial primers (357 F and 806 R) with a two-step amplicon library built on the Novaseq platform.

### Statistical analysis

Continuous variables are expressed as the mean ± standard deviation, and categorical variables are expressed as numbers and percentages. We tested the homogeneity of variances using Levene’s test. Variables were compared with two-tailed *t* tests or analysis of variance (ANOVA) if normally distributed and with the nonparametric Mann–Whitney *U* test if assumptions of normality or homoscedasticity were violated. We used the area under the receiver operating characteristic (ROC) curve (AUC) to quantify the model’s diagnostic performance for exploring the ability of SCFAs to distinguish ET patients from HCs or PD patients. To examine the associations between SCFAs and clinical severity, we used Spearman correlation analysis. Statistical analysis was performed using SPSS software (version 22.0; SPSS Inc., Chicago, IL), and the significance levels, including *P* value and FDR-P, were set at 0.05 (two-tailed).

The 16 S sequences were analyzed by using a combination of software Trimmomatic (version 0.35), Flash (version 1.2.11), UPARSE (version v8.1.1756), mothur (version 1.33.3) and R (version 3.6.3). The raw 16 S rRNA gene data were processed to form operational taxonomic units (OTUs) at 97% identity using UPARSE. Taxonomy was assigned using Silva 128 as the reference database. The genus level of the relative abundance data was selected for further analysis. Linear discriminant analysis (LDA) effect size (LEfSE) analysis was used for between-group comparisons (ET vs. HC, ET vs. PD) with an alpha cutoff of 0.05 and an effect size cutoff of 2.0. The discriminant genera identified by LEfSE analysis were further used for the Spearman correlation analysis of SCFAs.

### Reporting summary

Further information on research design is available in the Nature Research Reporting Summary linked to this article.

## Supplementary information

Supplementary materials

Reporting Summary

## Data Availability

The original 16 S sequencing data were deposited in the National Center for Biotechnology Information (NCBI) BioProject database (SRP438900: PRJNA974928) with an URL of https://www.ncbi.nlm.nih.gov/Traces/study/?acc=SRP438900&o=acc_s%3Aa. Other relevant data are available from the corresponding author upon reasonable requests, such as scientific cooperation and academic exchanges with complete research design. Any form of data sharing with third parties without our consent is not allowed.

## Code availability

Only open-source code from a combination of software Trimmomatic (version 0.35), Flash (version 1.2.11), UPARSE (version v8.1.1756), mothur (version 1.33.3) and R (version 3.6.3) were used, using either the default settings or the settings stated in the “Methods” section. Further information for clarification is available from the corresponding author on reasonable request.
