# Supplementary material for: Association of fecal short-chain fatty acids with clinical severity and gut microbiota in essential tremor and its difference from Parkinson’s disease
Source: NPJ Parkinsons Dis. 2023 Jul 17;9:115. doi: 10.1038/s41531-023-00554-5 (PMC10352256; doi:10.1038/s41531-023-00554-5)
Supplement: Supplementary file 1 — Supplementary materials [file 41531_2023_554_MOESM1_ESM.pdf]

Supplementary Materials

Supplementary Table 1 Comparisons of the fecal levels of SCFAs between groups

Supplementary Tables 2 Demographics and characteristics of early ET, PD and HC

Supplementary Table 3 Significant correlations between fecal levels of SCFAs and clinical features

Supplementary Table 4 Correlation between disease duration and SCFAs within ET and PD groups

Supplementary Table 5 Correlations between fecal levels of SCFAs and gut microbiota at genus level

Supplementary Figure 1 Between-group differences in fecal levels of SCFAs among early ET, PD and HC.

Supplementary Table 1 Comparisons of the fecal levels of SCFAs between groups

SCFAs（ug/g）	HC (n=35)	ET (n=37)	PD (n=37)	P value	P^1	P^2	P^3
Propionic acid *	1128.974 ± 521.506	861.578 ± 407.738	843.136 ± 406.855	0.023	0.023	0.013	0.837
Acetic acid *	1934.868 ± 838.117	1502.251± 516.241	1474.588 ± 479.384	0.039	0.054	0.016	0.620
Butyric acid *	1220.313 ± 760.868	755.181 ± 498.197	812.151 ± 497.269	0.020	0.007	0.041	0.508
Isovaleric acid *	104.831 ± 75.887	82.540 ± 52.301	121.107 ± 67.814	0.045	0.375	0.123	0.014
Valeric acid	181.789 ± 149.538	118.300 ± 97.843	124.106 ± 91.722	0.223	0.089	0.248	0.579
Caproic acid	41.298 ± 90.697	13.629 ± 25.932	21.474 ± 52.564	0.253	0.119	0.197	0.786
Isobutyric acid *	106.934 ± 60.978	76.690 ± 42.076	113.028 ± 54.122	0.015	0.040	0.479	0.005

P^1: HC vs. ET, P^2: HC vs. PD, P^3: ET vs. PD

ET, essential tremor; PD, Parkinson's disease; HC, healthy control; SCFAs, short-chain fatty acids.

* indicates significant group difference.

Supplementary Tables 2 Demographics and characteristics of early ET, PD and HC

	HC (n=35)	ET (n=16)	PD (n=33)	P value
Male (n, %)	19 (54.3%)	6 (37.5%)	16 (48.5%)	0.538
Age (y)	63.086±7.834	61.938±6.688	61.849±4.147	0.695
BMI (Kg/m^2)	24.145±3.107	23.825±2.834	23.582±1.843	0.676
Disease duration (y)	/	2.938±0.250	1.368±0.886	<0.001

ET, Essential Tremor; PD, Parkinson's Disease; HC, Healthy Control; BMI, Body Mass Index.

Supplementary Table 3 Significant correlations between fecal levels of SCFAs and clinical features

SCFAs (ug/g)	Clinical measurements	R	P value
Propionic acid	Wexner	-0.22988	0.01619
	SCOPA-AUT	-0.23552	0.043379
Acetic acid	Wexner	-0.20951	0.028779
Caproic acid	MDS-UPDRS	0.33529	0.042495
Isobutyric acid	FTM	-0.34868	0.03443
Isovaleric acid	FTM	-0.42068	0.009526
	TETRAS	-0.38173	0.019736

SCFAs, short-chain fatty acids; MDS-UPDRS, movement disorder society sponsored version of the unified Parkinson's disease rating scale; SCOPA-AUT, scale for outcomes in Parkinson's disease for autonomic symptoms; FTM, Fahn-Tolosa-Marin clinical rating scale for tremor; TETRAS, tremor research group (TRG) essential tremor rating assessment scale.

Supplementary Table 4 Correlation between disease duration and SCFAs within ET and PD groups

SCFA (ug/g)	ET disease duration (y)		PD disease duration (y)	
	**R**	**P value**	**R**	**P value**
Propionic acid	0.051	0.766	-0.037	0.826
Acetic acid	-0.225	0.182	-0.040	0.815
Butyric acid	-0.235	0.161	0.047	0.782
Isovaleric acid	-0.035	0.839	-0.017	0.920
Valeric acid	-0.068	0.690	-0.196	0.246
Caproic acid	0.008	0.962	-0.125	0.462
Isobutyric acid	-0.014	0.936	0.000	1.000

ET, essential Tremor; PD, Parkinson's disease; SCFAs, short-chain fatty acids.

Supplementary Table 5 Correlations between fecal levels of SCFAs and gut microbiota at genus level

	Genus	SCFAs (ug/g)	R	P value	FDR adjusted P value
**ET vs. HC**	↓Faecalibacterium*	Butyric acid	0.408	<0.001	0.014
	↓Lactobacillus	Butyric acid	0.283	0.016	0.096
	↓Catenibacterium*	Propionic acid	0.327	0.005	0.045
		Butyric acid	0.374	0.001	0.022
		Isobutyric acid	0.329	0.005	0.045
	↓Howardella	Propionic acid	0.242	0.041	0.163
	↓Raoultella	Propionic acid	0.249	0.035	0.158
	↓Candidatus Arthromitus	Isobutyric acid	0.302	0.010	0.072
	↑Stenotrophomonas	Isobutyric acid	-0.250	0.034	0.158
**ET vs. PD**	↑Lachnoclostridium	Isovaleric acid	-0.238	0.041	0.295
		Isobutyric acid	-0.257	0.027	0.295

ET, essential tremor; PD, Parkinson’s disease; HC, healthy control; SCFAs, short-chain fatty acids.

* indicates significance after FDR correction.

Supplementary Figure 1

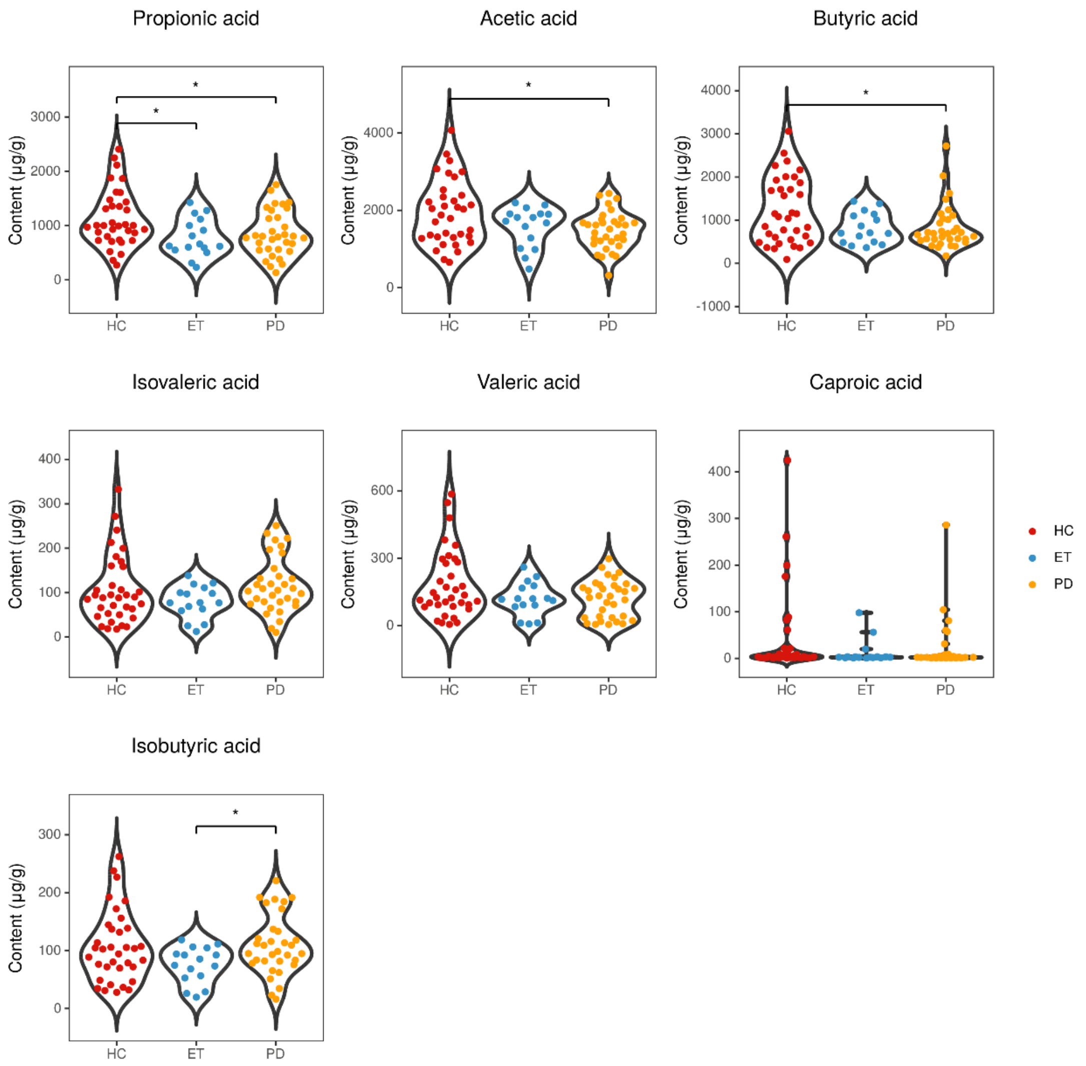

Supplementary Figure 1 Between-group differences in fecal levels of SCFAs among early ET, PD and HC. ET, essential tremor; PD, Parkinson’s disease; HC, healthy control, SCFAs, short-chain fatty acids. Significant differences are indicated by * $p < 0.05$.
